# Formative process evaluation of a guideline-driven process for improving the cultural responsiveness of alcohol and drug treatment services

**DOI:** 10.1186/s12913-021-06367-7

**Published:** 2021-04-15

**Authors:** Sara Farnbach, Julaine Allan, Raechel Wallace, Alexandra Aiken, Anthony Shakeshaft

**Affiliations:** 1 National Drug and Alcohol Research Centre UNSW, Sydney, NSW 2052 Australia; 2 School of Health and Society, University of Wollongong, Wollongong, NSW 2500 Australia; 3 Network of Alcohol and Drug Agencies, Woolloomooloo, NSW 2011 Australia

**Keywords:** Cultural responsiveness, Aboriginal and Torres Strait islander, Drug and alcohol, Service delivery, Implementation, Feasibility

## Abstract

**Background:**

To improve Australian Aboriginal and Torres Strait Islander people’s access to, and experience of, healthcare services, including Alcohol and other Drug (AoD) treatment services, principles and frameworks have been developed to optimise cultural responsiveness. Implementing those principles in practice, however, can be difficult to achieve. This study has five aims: i) to describe a five-step process developed to operationalise improvements in culturally responsive practice in AoD services; ii) to evaluate the fidelity of implementation for this five-step process; iii) to identify barriers and enablers to implementation; iv) to assess the feasibility and acceptability of this approach; and v) to describe iterative adaptation of implementation processes based on participant feedback.

**Methods:**

Participating services were 15 non-Aboriginal AoD services in New South Wales, Australia. Implementation records were used to assess the implementation fidelity of the project. Structured interviews with chief executive officers or senior management were conducted, and interview data were thematically analysed to identify project acceptability, and the key enablers of, and barriers to, project implementation. Quantitative descriptive analyses were performed on the post-implementation workshop survey data, and responses to the free text questions were thematically analysed.

**Results:**

A high level of implementation fidelity was achieved. Key enablers to improving culturally responsive practice were the timing of the introduction of the five-step process, the active interest of staff across a range of seniority and the availability of resources and staff time to identify and implement activities. Key barriers included addressing the unique needs of a range of treatment sub-groups, difficulty adapting activities to different service delivery models, limited time to implement change in this evaluation (three months) and the varied skill level across staff. The project was rated as being highly acceptable and relevant to service CEOs/managers and direct service staff, with planned changes perceived to be achievable and important. Based on CEO/management feedback after the project was implemented at the initial services, several improvements to processes were made.

**Conclusion:**

The operationalisation of the five-step process developed to improve cultural responsiveness was feasible and acceptable and may be readily applicable to improving the cultural responsiveness of a wide variety of health and human services.

**Supplementary Information:**

The online version contains supplementary material available at 10.1186/s12913-021-06367-7.

## Introduction

There is global concern about the impact of drug and alcohol use on health and wellbeing. The United Nations sustainable development goals include a target to strengthen the treatment of harmful substance use [1]. In Australia, enduring impacts of colonisation and racism have included Aboriginal and Torres Strait Islander peoples (hereafter referred to as Aboriginal) experiencing a disproportionate amount of harm from substance use [2]. To improve Aboriginal people’s access to, and experience of, healthcare services, including substance treatment programs, principles and frameworks have been developed to optimise cultural responsiveness (e.g. [3]). Cultural responsiveness is an ongoing process of adapting systems, services, and practice to fit with user preferences [4]. Although non-Aboriginal (mainstream) health services and clinicians may aim to provide health care that is culturally responsive to Aboriginal people, implementing its key principles can be difficult to achieve in practice [5].

Research to improve cultural responsiveness is in its infancy [6]. Key principles of culturally responsive practice for alcohol and other drug treatment services have been identified, including promoting client choice, facilitating community engagement and providing person-centred practice, which facilitate both best practice and an organisational culture which is inclusive and culturally responsive to all clients’ needs [7]. Nevertheless, barriers to practice improvement have also been identified at both the organisational-level, including leadership and organisational processes [8, 9] and the individual clinician-level, including a lack of knowledge and an attitudinal resistance to change [10]. A key hypothesis is that these barriers to implementing the principles of cultural responsiveness exist because there is a lack of clarity about how these principles can be operationalised [11].

Operationalising the principles of culturally responsive practice will require best-evidence implementation strategies [12]. Current evidence shows that since the provision of treatment guidelines, policies and training are ineffective on their own [13, 14], active learning processes are required to meaningfully engage key services’ staff in the process of change [15, 16]. The implementation process should be designed to engage staff in determining both what changes to make and how those changes should be implemented [16]. That flexibility is critical because there is evidence that different services will have different levels of cultural responsiveness at any one point in time [17] and because the approach to adopting more culturally responsive practice will need to be tailored to the individual circumstances of different services [16, 18–21].

This study has five aims: i) to describe a five-step process developed to operationalise improvements in culturally responsive practice; ii) to evaluate the level of implementation fidelity for this five-step process; iii) to identify barriers and enablers to implementation; iv) to assess the feasibility and acceptability of this approach; and v) to describe the process of iteratively adapting implementation processes based on participant feedback.

## Methods

The project, including the evaluation, was funded by a consortium of Primary Health Networks (PHNs) in New South Wales (NSW) to establish cultural responsiveness guidelines for non-government alcohol and drug services (hereafter NGO AoD services). These guidelines are not intended to replace the provision of services from specialist Aboriginal AoD services or community-controlled healthcare services, but to provide guidance to enhance the cultural responsiveness of non-Aboriginal or mainstream services. The project was developed and implemented using the principles of community-based participatory research [22] and involved several distinct phases, the design, methodology and participants in each phase are described in Table 1.

**Table 1:** Description of design features and participants in each phase of the project

Project phase	Participants, design and methods
Phase 1: Establishment of an Aboriginal advisory group	Aboriginal advisory group was established to advise and support the development and implementation of the guideline and comprised of Aboriginal members selected by expressions of interest submitted to the Aboriginal Drug and Alcohol Network (ADAN), invited representatives of the Aboriginal Health and Medical Research Council of NSW (AHMRC), and service providers selected by the project team. Advisory group members were encouraged to provide feedback and suggestions for improvement throughout the entire project [23].
Phase 2: Co-design of the five step-process	A five-step process was developed to identify, operationalise and measure improvements in culturally responsive practice in NGO AoD services. The process is further described in the results, but includes the following steps: 1. Development of a best-practice guideline 2. Baseline audits of participating services 3. Audit feedback to participating services 4. Guideline implementation workshops with participating services 5. Follow-up audits of participating services and audit feedback to services
Phase 3: Development of a best-practice cultural responsiveness guideline	A best-practice guideline that describes key elements of culturally responsive service delivery in non-Aboriginal NGO AoD treatment services was developed using the principles of community-based participatory research, aiming to empower services to make changes relevant to their local context and priorities, while making use of their existing strengths [22]. The guideline development and contents are described in the results section.
Phase 4: Recruitment of AoD services	Non-Aboriginal/mainstream NGO AoD services were invited by the commissioning PHNs, to participate in the implementation and evaluation of the guidelines. Of 17 services expressing interest, 15 chief executive officers (CEOs) or senior managers consented for their service to participate (88%) (hereafter referred to as participating services). Participating services included a variety of AoD service types/delivery models including residential rehabilitation (*n* = 3), day programs (*n* = 2), centre-based counselling and support (*n* = 3), outreach counselling and support (*n* = 4), groupwork and phone support (*n* = 1) and group or individual youth services (*n* = 2). Services varied in size from small volunteer-based to large national organisations. However, only one program within each service was audited. The largest program employed 20 staff and the smallest employed one part-time youth worker. The average number of employees in the audited programs was eight. Service participation was voluntary, and services did not receive financial incentives for participation in the evaluation. However, we note that the participating services do also receive funding from the PHNs that funded the project.
Phase 5: Implementation of the guidelines in participating services	After the development of the guideline (step 1), the remainder of the five-step process (steps 2 through 5) was implemented in participating services using a cluster randomised stepped-wedge design, with clusters based on the PHN district/geographic region (*n* = 6). The project schedule allowed three months between the baseline and follow-up audits. Key direct service/client facing staff working at participating services were nominated by management to attend the audits and implementation workshop. Services were encouraged to include CEOs or senior managers in the audits and implementation workshops so that they could contribute their detailed knowledge about services’ processes and policies, and so that staff with the capacity to decide and enact service level changes would be present to increase the likelihood that planned activities were implemented into services.
Phase 6: Mixed methods evaluation of the implementation and feasibility of the five-step process	A mixed methods approach was used to evaluate the fidelity of project implementation and assess the feasibility of the project. Data collection is detailed in the data collection and analysis section, but briefly, included: • Implementation records • Semi-structured interviews with service CEOs/managers • Post-implementation workshop surveys with direct service and management staff Participation in evaluation activities including interviews and surveys was voluntary and participants provided informed consent.

### Data collection and analysis

#### Implementation records

Throughout the project, a tracking document was maintained by members of the project team (RW, JA, SF) to establish the number and timing of services completing each component of the project. The essential components of the project to be delivered at each service included: baseline audit, provision of written audit feedback, implementation workshop and action plan, and follow-up audit.

#### Interviews with CEO or senior management

Semi-structured telephone interviews regarding enablers of, and barriers to, implementation of practice change were conducted with CEOs/managers of participating services after the baseline and follow-up audit. An interview guide was followed, which aimed to capture interviewee perspectives of audit outcomes; priorities for the implementation workshop (in first interviews only); feedback on the auditing process; preferences for ongoing development of cultural responsiveness, and perceptions about changes arising from the project (in second interviews only). Interviews were conducted by a researcher independent of the participating services (SF) digitally recorded and transcribed verbatim. A report summarising the priorities identified by the CEO/manager for the implementation workshop was provided to the project team (RW, JA) to assist with planning the implementation workshops. Interview data were thematically deductively analysed to identify enablers, barriers, and acceptability of the project using NVivo 12 [24].

#### Implementation workshop feedback survey

At the end of the workshop, the project team provided participants with a link to a brief online anonymous 24 item survey including questions about the participants’ role, reasons for attending and perceptions of the audit and workshop (see Additional file 1: Supplementary Appendix page 1 for a list of items). The survey was hosted on the online survey platform REDCap and administered by the evaluation team. Survey participants included staff from both direct service (*n* = 25) and management roles (*n* = 10), together representing approximately one third of all staff employed by the programs included in the project. To differentiate between direct service staff and CEO/management perspectives, only the responses from the direct service staff are described here. Simple descriptive analyses (e.g. frequencies) were performed using Excel [25], and responses to the free text questions were thematically analysed using NVivo 12 [24].

#### Iterative adaptation of the project components during the project implementation

To iteratively adapt the project in response to feedback from CEOs/managers obtained via interviews, the project team reviewed responses to identify feedback or potential improvements around project processes, acceptability, and relevance.

### Ethics

Service CEOs/management provided informed consent for services to participate in audits and participants (CEOs, managers, and staff) completed individual informed consent prior to taking part in interviews, workshops, and/or surveys. Ethical approval was provided by the AHMRC [#1487/19] and UNSW Sydney Human Research Ethics Committees [REC/16/CIPHS/46].

## Results

### A five-step process for operationalising improved cultural responsiveness

A five-step process to operationalise improvements in culturally responsive practice was devised, implemented, and evaluated using a program logic framework (see Additional file 2: Supplementary Appendix page 2 for the program logic).

#### Step 1: development of a best-practice guideline

A best-practice guideline that describes key elements of culturally responsive service delivery in non-Aboriginal AoD treatment services was developed [7]. The development of this guideline was led by an Aboriginal researcher with experience working with AoD services (RW). Extensive consultation was undertaken with Aboriginal community members in NSW including AoD clients/consumers, and with the project Advisory Group (which included senior Aboriginal AoD clinicians), to identify how they wanted AoD services to be delivered to Aboriginal people. Next existing guidelines detailing ways of working with Aboriginal people from health, community services, education and natural resource sectors and government departments were reviewed (*n* = 80) and strategies consistently reported in each of them were selected. This information was synthesised with feedback from the consultation into 6 themes and these themes operationalised into 21 action areas that describe key elements of culturally responsive service delivery in non-Aboriginal NGO AoD services. The themes and action areas are outlined in Table 2 and presented fully in the guideline document [7], available at https://www.nada.org.au/resources/alcohol-and-other-drugs-treatment-guidelines-for-working-with-aboriginal-and-torres-strait-islander-people-in-a-non-aboriginal-setting/.

**Table 2:** Summary of guideline themes and action areas

Theme	Action Areas
1: Creating a welcoming environment	*A welcoming greeting:* There is process for welcoming Aboriginal clients in a respectful manner and to introduce them to the program/service
	*A welcoming physical environment:* The design and layout are welcoming to Aboriginal people in the setting where most client contacts occur
2: Service delivery	*Service delivery:* Flexible and culturally informed service delivery practices are used
	*Referrals and assessments:* Immediate triage options are available
	*Direct practice:* Staff can provide examples of the ways they work therapeutically with Aboriginal people that is culturally competent
3: Voice of the community	*Community consultation and engagement to develop relationships:* Recent example of community engagement activity to develop relationships with Aboriginal people
	*Staff involvement with engagement activities:* Staff are familiar with community engagement processes
	*Local history and protocols:* Local protocols are reflected in practice and/or policy
4: Engagement with Aboriginal organisations and workers	*Working with organisations and workers:* Current connections with Aboriginal organisations and/or workers (referral pathways, shared work arrangements or relationships)
	*Collaborative Projects:* Current collaborative project with Aboriginal organisation or workers
	*New service/program establishment:* New service/program is established in consultation with Aboriginal community
5: Capable staff	*Staff knowledge and skill assessment:* Process to get feedback on and review the cultural competence of staff
	*Clinical/practice supervision around working with Aboriginal people:* Staff supervision includes feedback and opportunities to develop skills around working with Aboriginal people
6: Organisation’s responsibilities	*Employment practices:* Aboriginal representation on interview panels
	*Identified positions:* There are positions identified for Aboriginal people and Aboriginal networks are used to advertise positions
	*Service induction/mandatory training:* Training materials include information about working with Aboriginal people and are developed/reviewed by an Aboriginal person
	*Support for Aboriginal staff:* Cultural support/mentors, cultural support or skill development opportunities available to Aboriginal staff
	*Training staff in working with Aboriginal people:* Processes to train and continue to develop staff skills around working with Aboriginal people
	*Organisation wide practices:* Service has current Reconciliation Action Plan (RAP) and it is being implemented into practice
	*Aboriginal representation on the service’s Board:* The service has an Aboriginal representative on the Board
	*Policies and procedures:* Processes for Aboriginal people to contribute to policy development relating to Aboriginal people

#### Step 2: baseline audits of participating services

Structured baseline audits of current culturally responsive practice, relative to the best-practice guideline, were implemented in each participating service. Prior to the baseline audit, services were sent information explaining the audit process, what the audit is about, who should attend, how long it will take, information about the audit report and options for follow-up activities. Audits were conducted by two trained auditors who were external to the service, with at least one auditor who was Aboriginal. Audits were conducted in the setting where the service is delivered and took between 90 min to 2 h. Service staff attending the audits included a service or program manager or team leader and service delivery staff. Having a range of staff attending assisted in gaining explanations of both direct practice and organisational approaches to working with Aboriginal people. Where possible, the same staff attended the follow-up audit. Only information collected on the day of the audit was included in the scoring.

#### Step 3: audit feedback to participating services

Individualised written feedback from the audit findings were provided to each of the participating services. This was provided as a report listing all guideline action areas, a rating for each area reflecting the level of evidence observed during the audit (limited, some, good or excellent) and recommendations for potential areas where improvements could be made. An excerpt from an example baseline report is included in the Additional file 1: Supplementary Appendix (page 3).

#### Step 4: guideline implementation workshops with participating services

Implementation workshops were held with key staff (CEOs/managers and direct service staff) from services to explain the guideline, review the written audit feedback, set goals for improvement, and develop an action plan (to operationalise their improvement goals). Specifically, staff from each service were invited to select three priority action areas for their service (guided by the recommendations in the audit feedback) to progress over the next three months. They then made a detailed action plan to implement change, tailored to their specific service (an example action plan is included in the Additional file 1: Supplementary Appendix, page 5).

#### Step 5: follow-up audits of participating services

Follow-up audits of services were conducted after three months to assess change in culturally responsive practices, following the same procedure as for the baseline audits. Services were again provided with individualised written feedback, which also included some observations of any changes that had occurred. An excerpt from an example follow-up audit report is included in the Additional file 1: Supplementary Appendix (page 7).

### The fidelity of the implementation of the five-step process

The five-step process was predominantly implemented as planned, with a high level of fidelity achieved for each of the five project components: a) development of guidelines; b) baseline audit of services; c) written feedback; d) staff attending implementation workshops; and e) follow-up audit of services. The guideline was published and provided to all participating services after the baseline audit. Twelve of the 15 participating services completed all four service-specific project components (b to e). One service completed (b) to (d), one completed (b) only, and one did not complete any of the project components (see Table 3). The main reasons for services not completing all the project components included staff turnover during the project or the project team was unable to contact service staff to arrange subsequent components.

**Table 3:** Implementation of each project component in participating services

Cluster*	Invited to participate (***N***)	Participating services at baseline (***N***)	Project component				Mean time between audits (weeks)
			A Baseline audit (***n***)	B Attended workshop (***n***)	C Completed action plan (***n***)	D Follow-up audit (***n***)	
**1**	3	2	2	2	2	1	16
**2**	2	2	2	2	2	2	15
**3**	2	2	2	2	2	2	23
**4**	3	3	2	2	2	2	16
**5**	5	4	4	3	3	3	19
**6**	2	2	2	2	2	2	17
**All services**	**17**	**15**	**14**	**13**	**13**	**12**	**18**

* Clusters based on PHN district/geographic region

Follow-up audits were scheduled to be completed 12 weeks after the baseline audit, however the mean time between audits was 18 weeks (range 14–28 weeks) (Table 3). Delays were predominantly due to scheduling commitments at services and staff availability. Of those with the longest delays, one service was re-scheduled due to a local bushfire (Cluster 3) and another was delayed due to unavailability over the Christmas holiday period (Cluster 5).

Interviews with CEOs/managers at baseline (*n* = 14) and follow-up audit (*n* = 12) showed that audit reports were received by all services that completed an audit and an interview. Staff and CEOs/managers were actively engaged in the project, with staff from 13 services attending implementation workshops (Table 3). Six CEOs/managers reported some uncertainty among some of their staff about the project requirements before the baseline audit (about the project’s background, expectations, scheduling, and next steps). These uncertainties were clarified during discussions at baseline audits. In the implementation workshop feedback survey, most direct service staff (23/25) reported that they were aware that the baseline audit had occurred at their service and over half (16/25) were aware of the outcome of the audit.

### Enablers and barriers to implementation

Thematic analysis of interview responses identified CEO/manager perspectives on barriers and enablers to implementing cultural responsiveness activities (Table 4). Enablers included aligning the timing of the project with setting up new services, having multi-level buy-in for the project and having resources/staff time available to support project activities. Barriers included limited funding and time available to complete planned activities, challenges hiring Aboriginal or culturally responsive staff, the need to balance the needs of varied population groups, difficulty adapting activities to different service delivery models and limited time to implement change.

**Table 4:** Common enablers and barriers to implementing culturally responsive activities reported by CEOs’ and managers’

Enablers	Description
Timing of project with service changes or setting up new programs	New services/programs or those undergoing internal changes (e.g. re-structuring, strategic planning, or developing/implementing Reconciliation Action Plans) were well positioned to implement changes to culturally responsive practice. *“The specific service that was being looked at is actually a new program for us and we’re still actually yet to officially start the program. So, it was very useful to actually have [the auditor] come in actually just before we actually commenced service delivery and actually look at where we are in terms of our … cultural intelligence and cultural competence … and where we’re at as a service, before we actually start commencing service delivery.” Manager, Service J*
Interest in the project from multiple levels within services	Buy-in from CEOs/managers and direct service staff who attended project activities led to a productive environment which supported action around culturally responsive practice. *“I have made sure that staff was able to network, because there was a large networking component in terms of the seeking integration with the local [Aboriginal] services, really setting up those relationships. So basically, I ensured that both on the frontline level as well as core coordinator level such as myself that multiple levels of staff were involved with that initiative.” Manager, Service K*
Resources/staff time available to progress activities	Staff had adequate time and funding with which to dedicate to activities supporting action around culturally responsive practice. *“I think it’s great to have somebody that’s dedicated to that work [cultural responsiveness] that could be doing it for us as well, because I think people just get caught up in the day-to-day and they get caught up in crisis, and those really, that work that requires time and for, and relationship building sometimes gets left aside unfortunately.” Manager, Service F*
**Barriers**	**Description**
Limited access to funding and time to progress activities	Funding was not readily available to support specific activities (developing resources, community engagement) or for positions which focused on work around cultural practice (including clinical, community engagement and project roles, particularly of dedicated roles for Aboriginal staff). It was sometimes challenging to allocate staff time to complete project activities around busy existing workloads and competing service demands. *“I think identified positions are really important and we know we need more identified positions. Just finding the funding for that is the difficult part.” Manager, Service H*
Challenges hiring Aboriginal staff or culturally responsive staff	Challenges hiring appropriately skilled staff to identified and non-identified positions, especially in rural/remote areas. Sometimes when roles were advertised, there were no Aboriginal staff applications for extended periods, or in other cases, applicants were over or under qualified. Sometimes managers decided not to hire people because they did have strong cultural skills, meaning that clinical positions took longer to fill. *“I’m always trying to get funding to get an identified Aboriginal worker. We advertise. We do advertise for frontline workers. We put in the advert that we really want Aboriginal people to apply and it’s open to Aboriginal people. We’ve put that in as a clause. Unfortunately, we’re not getting much.” Manager, Service I*
The need to balance the needs of varied population groups	Services often had clients from multiple ethnic, cultural, and religious backgrounds, which required them to be responsive. This resulted in some services having a limited capacity to tailor specific workflows and processes to Aboriginal clients. *“I think for our organisation … we span over quite a diverse geography … so we actually have services on lots of different country. What we’re finding to be somewhat difficult is how do we as an organisation support cultural competency from an organisational level, to then actually pay respect to the nuances of the different communities that we’re in.” Manager, Service L*
Difficulty adapting activities to different service delivery models	Services differed with respect to their delivery models, geographical locations, and organisational size, which meant that activities had to be adapted or in some cases, were not feasible for specific settings. Some larger state and national organisations had internal processes which required longer timeframes to implement activities, and in some instances, proposed activities were not feasible because of these processes/policies (e.g. including Aboriginal board members). “*… the other activity that we’d planned was around trying to have a stronger connection with the local Aboriginal community. Again, that’s challenging I think … because all of our services are outreach services.” Manager, Service B*
Limited time (3 months) to implement change	The timeframe was too short to show sustained change or implement activities, such as developing new relationships with Aboriginal representatives. The 3-month follow-up audit was useful because it motivated staff to continue working towards achieving their planned activities before the follow-up audit. *“I had to manage all the staff leave and annual holidays, Christmas itself, so even though we had three months, it really, when you shook it down, it was more like two months but it was broken up over, it was all very disjointed.” Manager, Service A*
Varied skill level across staff	Some staff had extensive skills working with Aboriginal people, others required additional time to develop their skills and knowledge. Providing training to staff sometimes slowed down implementation. *“When you’re getting these things, you don’t know what you don’t know, so it’s really difficult. You know, they didn’t know about … Land Councils, they didn’t know about how to engage with elders, they didn’t know that you can get flags from the local federal minister, from their office. So, the little things that you know about, they didn’t know that there was a cycle of the Aboriginal model of the cycle of behaviour change. So, all those things that were sort of just bread and butter when you’re working in AMS [Aboriginal Medical Service], when you step over to mainstream, people have just not been exposed to it.” Manager, Service F*

### Feasibility of the project - acceptability and staff perceptions

#### Acceptability to CEOs/managers

The project was reported to be acceptable by all (100%) CEOs/managers who completed interviews after the baseline (*n* = 14) and follow-up audits (*n* = 12). Most reported that it had benefit to the services and to themselves, and that implementing cultural responsiveness activities was an important part of their work and a priority at their service. Many reported that their service had a focus on cultural responsiveness before the project began.

#### CEOs/managers’ perspectives of the workshop and audits

Thematic analysis of interviews with CEOs/Managers at follow-up (n = 12) identified several major themes as outlined in Table 5.

**Table 5:** CEOs’ and managers’ perspectives of the project

Theme	Description and quotes
Services benefited from participating in the project	Services and staff built capability and skills around specific activities involved with culturally responsive service delivery. Many staff reported that they found the project resources useful, particularly audit outcome reports, the Guideline, and the action planning tool (completed by staff in the implementation workshop to plan actions over the subsequent three months). “*The Guidelines are useful, but I would say the audit report was even more useful … having an organisation actually come in actually go, “this is where you’re doing well. These are the areas you can improve on,” I think that’s really very valuable. So, moving forward, I would suggest that we’re probably going to look at the recommendations in the audit report rather than the Guidelines.” Manager, service J*
Audits and audit outcome reports prompted change	Managers reported that completing the audits and receiving the audit outcome reports provided them with new insights and ideas about how cultural responsiveness principles can be applied in practice. Sometimes, staff members reported that they devised and applied new strategies around cultural responsiveness before they attended the implementation workshop and completed action planning. *“There’s been two new clients since [the audit] last week that are Aboriginal, and [staff] have started conversations, good policy conversations about the greetings, the welcoming [environment]...” Manager, service D*
There was personal benefit from the project	Many staff reported benefits arising from learning new skills/knowledge as part of the project, or from spending time working on a different project to their useful duties. *“From my end as a clinician, I could look at it as professional development, because there are things I didn’t learn at university, I didn’t learn in placement, but now I’m equipped with these resources that I’ve passed on to the team.” Manager, service J*
There is keen interest among staff around implementing cultural responsiveness	Delivering culturally responsive care was viewed as an important aspect of service delivery. *“It’s been a really positive for us, and I think it’s given us a really good framework of where we need to step up and what we can be doing a little bit more … and what things will be looking like for us to move forward to be working in a safe place for our clients.” Manager, service L*

#### Acceptability and perceptions of direct service staff regarding the workshops

Twenty-five direct service or client-facing staff members from participating services attended implementation workshops and completed the survey. Participants agreed that the logistics of workshops were well organised (100%) and reported high satisfaction with the workshops (92% satisfied/very satisfied). Participants were also satisfied with the content and delivery of the workshops, with all (100%) agreeing that the workshops were well facilitated, the aims were clearly explained, the content was relevant and useful, and the workshop activities worked well, and 96% also agreeing that the planning tool was useful. All (100%) participants reported that they felt they had a clear plan after the workshop about how to change the cultural responsiveness of their service and most felt that they had the resources and support available to implement change (92%).

### Iterative adaptation of the project components during the project implementation

Interview participants (CEOs/managers) were given the opportunity to provide suggestions for how the project could be improved in each interview. Suggestions were discussed among the project and evaluation team and the following components of the project were subsequently updated. Suggestions were mostly made by those interviewed from services audited early in the project. After these changes were made, no further substantive feedback was provided regarding improvements to the project.

1. Information provided to CEOs/managers at the beginning of the project emphasised the recommendation that a CEO/manager attend the audits, and clearly described the audit process to indicate that only information provided on the day of the audit would be included in the audit rating and report. This recommendation was made to ensure staff with knowledge around services’ processes/policies attended the audit, so information they provided could be included in the audit outcome report and considered during rating allocation. Due to the scheduling commitments of the project, the audit only captured information provided at the time of audit, and staff were not able to comment and provide additional information after the audit outcome report was provided to services.
2. The audit report was restructured and developed during the project. Reports were shortened and wording was revised. Numerical ratings were removed from reports. The updated version used the words ‘*limited, some, good or excellent’*, replacing the rating of 0, 1, 2 or 3. Changing the reporting around the rating system underpinning the audit system provided service staff with encouraging feedback, with the aim of motivating them to take on and implement the audit feedback.

## Discussion

The project successfully developed a best-practice guideline that describes key elements of culturally responsive service delivery in non-Aboriginal AoD treatment services [7] and implemented these guidelines in 15 NGO AoD services. This process was led by an Aboriginal researcher and involved extensive consultation with an Aboriginal advisory group, community members, and AoD service providers and organisations [26–28]. Overall a high level of implementation fidelity was achieved, with most participating services completing all components and both management and direct service staff demonstrating a high level of engagement with the process. Delays in completing follow-up audits was the only significant departure from the planned project implementation. Feedback from CEOs/managers indicated that the short time between audits was a barrier to the realization of more complex change activities. Future work using the five-step process devised for this project may benefit from extending the time between audits. This would provide more time for services to implement planned actions and for any changes implemented to have an impact on client outcomes.

The resources and processes developed through this project including the guidelines, audit tools, action plans and implementation workshops are feasible to use and highly acceptable to management and direct service staff at the participating services and the participatory research approach resulted in improvements in the delivery of the five-step process. While the guideline was initially thought to be the driving force underpinning the process, the importance of the other steps was quickly realised. The auditing of services, providing individualised feedback and assistance in creating action plans appeared to be key in helping services to improve cultural responsiveness. Direct service staff rated the project resources (feedback reports and action plans) as particularly useful tools for implementing change activities. As has been previously identified [15, 16], active learning processes in the implementation workshops appeared to be important in engaging both management and direct service staff and in operationalising the cultural responsiveness concepts into concrete activities.

An important strength of the project is the flexibility with which the guidelines can be implemented within individual services [19]. The recommendations in the audit reports personalise the guidelines for individual services and staff can then use the action plan to focus on those change activities identified as important and achievable for their individual services. This approach of intervening using a balance of standardised and flexible components is supported by existing evidence [16, 18, 19] and appears to be well suited to use in NGO AoD treatment services. The commitment to, and enthusiasm for the concept of cultural responsiveness from staff across multiple levels was identified as an enabler for change by CEOs/managers, consistent with previous research [16, 29]. The high level of participation and engagement from both direct service staff and CEOs/managers suggest that staff are actively aiming to enhance culturally responsive service delivery in the NGO AoD service setting. In particular, engagement of senior staff with detailed knowledge about services’ processes/policies related to cultural responsiveness and the capacity to decide and enact service level changes, appeared to be important in ensuring that planned activities were implemented into services.

Limitations to note include the relatively small number of self-selected services involved in the project, though they do represent a wide geographic and sociodemographic area of NSW and a variety of service types/delivery models. Self-selection bias is possible; services may have had pre-existing interest cultural responsiveness and/or some resources to dedicate to the process. Such a high level of engagement may not be observed in other services, given that organisational climate is a known enabler of change [16]. CEOs/managers selected directed service staff to participate in the project and not all staff at participating services were involved in the project, thus the responses described in this paper may not reflect the views of the wider service staff. There is limited information on the acceptability to those services that did not complete all components, however, drop out appears to be related to staff turnover and scheduling issues, rather than dissatisfaction with the project.

A significant barrier to actioning changes was that services lacked resources including funding and staff time with which to implement changes. Establishing cultural responsiveness practice as a routine continuous quality improvement (CQI) cycle in services might help to overcome this, and other barriers, to improvement [30]. Repeated CQI cycles have been shown to be an effective process for systems change in Aboriginal health services in a study examining diabetes care, with flow on improvements in key health indicators [31, 32]. Using this approach, services aim to incrementally improve practices over time, whilst tracking improvements through routinely collected data and regular auditing [32]. Routine evaluation frameworks could be seamlessly embedded into service delivery at low-cost by better use of administrative data that are already routinely collected. This approach would allow services time to seek out funding or resources or to schedule change activities in line with funding or other service changes. A longer-term follow-up audit of participating services is currently underway, including the identification and assessment of routine service delivery data, to evaluate the impact of the guidelines on cultural responsiveness. Future plans include working with services to develop pragmatic methods for embedding the process as a CQI, or at least ensuring that the process of improvement continues regardless of staff turnover or other organisational change. While the current project does not directly address the feasibility of scaling up the intervention, the resources developed could feasibly be utilised as a resource for all mainstream AoD services and the external auditing of mainstream services is potentially a service that could be delivered by an Aboriginal AoD organisation.

## Conclusion

This project developed a cultural responsiveness guideline following the principles of community engagement, choice, and person-centred practice. The results support the use of a pragmatic and participatory evaluation approach using a standardised core intervention and flexible components that allowed different types of services to prioritise different aspects of cultural responsiveness and to implement solutions that suited their unique needs and strengths. The operationalisation of the five-step process developed to improve cultural responsiveness was feasible and acceptable and is readily applicable to improving the cultural responsiveness of a wide variety of health and human services.

## Supplementary Information

**Additional file 1: Appendix A.** Implementation workshop feedback survey items. **Appendix B.** Evaluation Framework and Program Logic - Project level. **Appendix C.** Example excerpt from a baseline service audit report.

## Data Availability

The datasets used and/or analysed during the current study are available from the corresponding author on reasonable request.

## Abbreviations

ADAN: Aboriginal Drug and Alcohol Network
AHMRC: Aboriginal Health and Medical Research Council of NSW
AoD: Alcohol and other drugs
CEO: Chief executive officers
CQI: Continuous quality improvement
NGO: Non-government organisation
NSW: New South Wales
PHN: Primary Health Network
