# Supplementary material for: Formative process evaluation of a guideline-driven process for improving the cultural responsiveness of alcohol and drug treatment services
Source: BMC Health Serv Res. 2021 Apr 15;21:352. doi: 10.1186/s12913-021-06367-7 (PMC8051070; doi:10.1186/s12913-021-06367-7)
Supplement: Supplementary file 1 — Additional file 1: Appendix A. Implementation workshop feedback survey items. Appendix B. Evaluation Framework and Program Logic - Project level. Appendix C. Example excerpt from a baseline service audit report. [file 12913_2021_6367_MOESM1_ESM.docx]

**Appendix A: Implementation workshop feedback survey items**

1. Please enter your role / job title:
2. Client-facing role: *yes/no*
3. Reason for attending:

*Workshop planning and organisation*

*(response options: strongly agree, agree, disagree, strongly disagree)*

1. The logistics and communication for planning the workshop were well executed e.g. arranging dates, location, support/funding provided
2. The workshop was well organised

*Workshop aims and content*

*(response options: strongly agree, agree, disagree, strongly disagree)*

1. The aims of the workshop were clearly explained
2. The overall content at the workshop was relevant and useful
3. The activities (video, planning sessions, group discussion) at the workshop worked well
4. The workshop sessions were well facilitated

*(response options: yes/no and (open ended)*

1. Did you find the content of the workshop challenging?
2. [If yes to 10] What did you find challenging about the workshop?

*Implementing the Guideline after the audit*

*(response options: yes/no and (open ended)*

1. Before today’s workshop, were you aware of the Cultural Competence Guideline audit that took place at your service?
2. Before today’s workshop, were you aware of the audit outcomes from your service?
3. Since the audit, are you aware of any changes at your service that relate to cultural competence?
4. [If yes to 14] What changes were made? were you involved with these changes?
5. [If yes to 14] Why did these changes occur?

*Implementing the Guideline after the workshop*

*(response options: yes, why?(open ended) / no, why?(open ended)*

1. After the workshop, do you have a clear plan about how to improve the cultural competence of your service?
2. Did you find the planning tool helpful to plan activities?
3. Do you have all the resources and support you need to implement the activities you planned today?
4. Do you have any suggestions for future workshops? *(open ended)*
5. Please indicate your current satisfaction with this workshop by selecting the appropriate number: *1: very unsatisfied, 2: unsatisfied, 3: okay/neutral, 4: satisfied, 5: very satisfied*
6. Any comments to explain your rating *(open ended)*

| **Appendix B: Evaluation Framework and Program Logic - Project level** | | | | |
| --- | --- | --- | --- | --- |
| **Research question:** **Was the guideline implemented in the nominated services (nominated by the PHNs) and did it change (improve) the cultural competence of services?** | | | | |
| **a. Assumptions underpinning project** | **b. Modification (main project activity)** | **c. Mechanisms of change** | **d. Project Outputs** | **e. Impact outcomes** |
| Some Aboriginal clients will access non-Aboriginal services  There is a lack of guidance for non-Aboriginal services around processes involved with culturally competent service delivery  Some Aboriginal clients who would access non-Aboriginal services are more likely to initiate and complete treatment if culturally competent care is available  Audits will identify existing practices around cultural competence  Staff will be able to describe compliance (or not) with key processes described in the Guideline (at audits)  Services will be able to achieve changes (improvements) to cultural competence through a range of 1-off or ongoing activities that relate to Guideline Themes | *Develop Guideline* | | | ***Primary outcome*:**  Change in cultural competence of services in Action Areas^ from the Guideline (audit rating post- verse pre-) ^d,g^  ***Secondary outcomes:***  A: A significant increase the proportion of Aboriginal people attending the service ^j^  B: A significant increase in the number of Aboriginal people who complete treatments ^j^  C: A significant increase the number of occasions of service made by Aboriginal clients ^j^  D: Change in cultural competence of services in all Themes from the Guideline (audit rating post- verse pre-) ^d,g^  **Data collection sources**:  ^a^ Guideline  ^b^ Implementation & Evaluation Log  ^c^ Pre-implementation workshop interview with CEO/Senior staff member nominated by the service to work on the project  ^d^ Baseline Audit  ^e^ Action plans developed by staff during the Implementation Workshop  ^f^ Implementation Workshop Feedback Survey (anonymous online survey)  ^g^ Follow up Audit  ^h^ Client Feedback (collected by services, where available)  ^i^ Post Implementation Interview with CEO/Senior staff member  ^j^ Minimum Data Set data – provided by NADA with permission from services |
|  |  | Streamlining particular processes according to sound practice will improve culturally competent service delivery | - Guideline finalised |  |
|  | *Baseline Audit* | | |  |
|  |  | Systematically reviewing service delivery against sound practices (the Guideline) will identify opportunities for improvements in cultural competence before the guideline is implemented at services | - Number of services* that participated in the baseline audit ^b^ |  |
|  |  |  | - Number of services with rating for each audit process (total = 21) ^d^ |  |
|  |  |  | - Staff members experiences with the baseline audit ^c^ |  |
|  |  |  | - Number of services that completed Pre-implementation workshop interview ^b^ |  |
|  | *Implementation workshops* | | |  |
|  |  | Co-designing a few key activities will lead to improved cultural competence through developing tailored activities | - Number of services that had 3 staff attended implementation workshop ^b^ |  |
|  |  |  | - Number of services that drafted a plan for 3 or more activities to improve cultural competence ^e^ |  |
|  |  |  | - Staff members experiences and attitudes with the Implementation Workshop ^f^ |  |
|  | *Follow up Audit* | | |  |
|  |  | Re-reviewing service delivery against sound practice (the Guideline) will identify changes in cultural competence, and opportunities for ongoing improvements | - Number of services that completed follow up audit ^g^ |  |
|  |  |  | - Changes in client feedback to service^#^ ^h^ |  |
|  |  |  | - Staff members experiences with the project (audits & implementation) ^i^ |  |
|  |  |  |  |  |

**Impact Outcome definitions:**

Cohort defined from MDS statistics and includes: ‘Aboriginal & Torres Strait Islander’ &

‘Aboriginal but not Torres Strait Islander Origin’.

Outcome A: proportion of cohort according to ‘Episode ID’

Outcome B: proportion of cohort according to 'Reasons for Cessation of Service'

Outcome C: proportion of cohort according to ‘Client ID’

Outcome D: change in total audit rating between pre- and post- audit

**Appendix C: Example excerpt from a baseline service audit report**

| **THEME 1 - CREATING A WELCOMING ENVIRONMENT** |
| --- |
| **1A: A WELCOMING GREETING**  **What does this action area mean?**  The service has a process to greet and welcome Aboriginal people when they arrive at the service that is consistently implemented across the service.  **What was the rating at our service?** There is **excellent** evidence of a welcoming greeting. The service has reception staff and clients are offered refreshments on arrival. Clients are then contacted by a drug and alcohol worker, who meets the clients at a place convenient for them. |
| **1B: A WELCOMING PHYSICAL ENVIRONMENT**  **What does this action area mean?** The physical environment where clients spend time has comfortable seating, has Aboriginal resources on display and can accommodate children.  **What was the rating at our service?** There is **excellent** evidence of a welcoming physical environment. The service’s outreach process results in staff meeting clients at a place convenient for them e.g. young people are often seen at school. Although schools may not have the ideal physical environment, they are a safe place for most young people. |
| **Action Areas 1A and 1B align with the Australian National Safety and Quality in Health Care standard: 1.33 Clinical Governance** |
|  |
| **THEME 2 - SERVICE DELIVERY** |
| **2A: FLEXIBLE SERVICE DELIVERY OPTIONS**  **What does this action area mean?** Service delivery is flexible around Aboriginal clients via providing services in a range of settings and using culturally relevant resources when working with Aboriginal clients.  **What was the rating at our service?** There is **limited** evidence of flexible service delivery options. Flexible service delivery options such as providing outreach or using culturally relevant resources when working with Aboriginal service users were not reported.  **How to do it?** There are opportunities to deliver services via outreach to Aboriginal services, working with other service providers and to use culturally relevant resources when working with Aboriginal clients. |
| **2B: IMMEDIATE ASSESSMENT AND REFERRAL OPTIONS**  **What does this action area mean?** There are immediate assessment and referral options that are tailored for Aboriginal people.  **What was the rating at our service?**  There is **some** evidence of immediate assessment and referral options that are tailored for Aboriginal people. The service will respond to a service user or potential service user within 24 hours. Crisis support numbers are provided. It can take up to two weeks to see a worker, but this is rare and usually a new service user will be seen within the first week.  **How to do it?** There are opportunities to continue to develop immediate assessment and referral options that are tailored for Aboriginal people. |
| **Action Area 2B aligns with the Australian National Safety and Quality in Health Care standard: *5.8 Comprehensive Care*** |
| **2C: DIRECT PRACTICE**  **What does this action area mean?** Staff can provide examples of direct practice strategies that are tailored be culturally sensitive to Aboriginal people.  **What was the rating at our service?** There is **good** evidence of staff using culturally sensitive direct practice strategies. Examples included the use of screening tools and the use of the Power, Threat, Meaning Framework and narrative approaches. However, it was not clear how widespread the use of these frameworks was in the service.  **How to do it?** There are opportunities to continue to tailor the direct practice strategies used by staff to be culturally sensitive to Aboriginal people. |

**Appendix D: Action plan template with example activity**

| **ACTION PLAN TEMPLATE: Cultural competence workshop - XX service** | | | | |  |  |  |  |
| --- | --- | --- | --- | --- | --- | --- | --- | --- |
| **Project lead** | **Billy** |  |  |  |  |  |  |  |
| **Action: What do we do? What resources are required?** | **Who is responsible?** | **Who do we involve?** | **What is the output?** | **What is the priority?** | **What is the status?** | **Start Date** | **End Date** | **Notes** |
| **Goal #1: Display Acknowledgement of Country at service (Example activity)** | | | | | | | | |
| Get management approval to display sign, speak with XX | Billy | CEO | Management approval received in writing | High | In progress | 18-Apr-19 | 18-Apr-19 | CEO has agreed in principle, will formalise on Thurs at meeting |
| Discuss at staff meeting and agree on location of sign at service | Sally | CEO & all staff | Discussed at staff meeting; location is agreed | High | In progress | 24-Apr-19 | 24-Apr-19 | Regular staff meeting |
| Make contact with ACCHO/Elders group | Billy | XX from ACCHO/LC | Meeting with ACCHO/Elders, advice received | High | In progress | 28-Apr-19 | 5-May-19 | Already in contact, will make formal request |
| Finalise wording/pictures for sign (review by ACCHO/Elders group) | Billy | XX from ACCHO/LC | Wording and map finalised, reviewed by ACCHO rep | Med | Not started | 5-May-19 | 15-May-19 |  |
| Find sign maker/contractor to make the sign | Admin | Admin & contractor | Contractor is identified, ready to go | Low | Not started | 5-May-19 | 8-May-19 | Need to put in request to admin |
| Identify budget for the sign | Maria | Finance dept | Budget is available | Med | Complete | 18-Apr-19 | 18-Apr-19 | CEO has approved - up to $400 |
| Get the sign made | Maria | Finance dept | Sign is completed |  |  |  |  |  |
| Put up the sign in agreed location | CEO | All staff | Sign is displayed | High | Not started | 30-May-19 |  | ?? Arrange a morning tea? Invite ACCHO reps? |
| **Goal #2:** |  |  |  |  |  |  |  |  |
|  |  |  |  |  |  |  |  |  |
|  |  |  |  |  |  |  |  |  |
|  |  |  |  |  |  |  |  |  |
|  |  |  |  |  |  |  |  |  |
|  |  |  |  |  |  |  |  |  |
| **Goal #3:** |  |  |  |  |  |  |  |  |
|  |  |  |  |  |  |  |  |  |
|  |  |  |  |  |  |  |  |  |
|  |  |  |  |  |  |  |  |  |
|  |  |  |  |  |  |  |  |  |
|  |  |  |  |  |  |  |  |  |

**Appendix E: Example excerpt from a follow-up service audit report**

| **THEME 3 – VOICE OF THE COMMUNITY** |
| --- |
| **Action area 3A includes two audit activities: 3AI and 3AII**  **AUDIT ACTIVITY 3AI: RECENT COMMUNITY ENGAGEMENT**  **What does this audit area mean?** In the last 12 months, there are regular and effective examples of the service engaging with Aboriginal community members or representatives from local Aboriginal Lands Councils or similar community organisations.  **What was the rating at our service at follow-up?** There is **good** evidence of recent community engagement. This is great improvement from the baseline rating of **some**!  **What has changed since baseline?** Examples given at the follow-up audit include:   - Meeting with local Aboriginal community agencies and contacting staff at these agencies - Staff are supportive of greater community engagement - Staff are learning more about local Aboriginal history with information from the University of Sydney   **How to do it?** There are opportunities to continue to increase engagement with Aboriginal staff, Aboriginal community members or representatives from local Aboriginal organisations. |
| **Action Area 3A aligns with the Australian National Safety and Quality in Health Care standard:** ***2.13 Partnering with Consumers*** |
| **AUDIT ACTIVITY 3AII: STAFF AWARE OF THE SERVICE’S ABORIGINAL COMMUNITY ENGAGEMENT ACTIVITIES**  **What does this audit area mean?** All staff are aware of the service’s engagement with Aboriginal community members or representatives from local Aboriginal community organisations.  **What was the rating at our service at follow-up?**  There is **some** evidence of staff awareness of service’s Aboriginal community engagement activities. At the baseline audit the rating was the same. Staff knowledge of community engagement strategies is inconsistent. The manager is well connected and knowledgeable about community engagement; however, knowledge does not appear to be widespread across the service.  **How to do it?** There are opportunities to continue to develop processes to inform staff about the service’s engagement with Aboriginal staff, Aboriginal community members or representatives from local Aboriginal organisations. |
| **3B LOCAL HISTORY AND PROTOCOLS**  **What does this action area mean?** The service has a process to build knowledge of local Aboriginal history, culture and protocols and to incorporate this knowledge into the service’s practices and policies. All staff are aware of the local Aboriginal history, culture and protocols.  **What was the rating at our service at follow-up?**  There is **good** evidence of the service incorporating knowledge of local Aboriginal history, culture and protocols into practices and policies. This is an improvement from the baseline rating of **some.**  **What has changed since baseline?** Staff described a growing understanding of why local information was important and plans to consult Elders have been made but not yet completed. This demonstrates an intentional approach to identifying or understanding local circumstances. Audit participants stated they have “go to” people in each community who provide them with local links, but they understand that is not a whole of community view.  **How to do it?** There are opportunities to continue to build knowledge of and incorporate local Aboriginal history, culture and protocols into the service’s practices and policies, and to inform all staff of the local Aboriginal history, culture and protocols. |
